# Leveraging community Wi-Fi and spaces for digital health use

**DOI:** 10.3389/fpubh.2024.1418627

**Published:** 2024-06-07

**Authors:** Erin M. Spaulding, Hailey N. Miller, Faith E. Metlock, Joyline Chepkorir, Chitchanok Benjasirisan, Melissa D. Hladek, Hae-Ra Han

**Affiliations:** ^1^Johns Hopkins University School of Nursing, Baltimore, MD, United States; ^2^Digital Health Innovation Laboratory, Ciccarone Center for the Prevention of Cardiovascular Disease, Division of Cardiology, Department of Medicine, Johns Hopkins University School of Medicine, Baltimore, MD, United States; ^3^Welch Center for Prevention, Epidemiology, and Clinical Research, Johns Hopkins Bloomberg School of Public Health, Baltimore, MD, United States; ^4^Center for Community Programs, Innovation, and Scholarship, Johns Hopkins University School of Nursing, Baltimore, MD, United States; ^5^Department of Health, Behavior, and Society, Johns Hopkins University Bloomberg School of Public Health, Baltimore, MD, United States

**Keywords:** health care disparities, health services accessibility, public health, internet access, telehealth, telemedicine, digital health

## Abstract

Digital health disparities continue to affect marginalized populations, especially older adults, individuals with low-income, and racial/ethnic minorities, intensifying the challenges these populations face in accessing healthcare. Bridging this digital divide is essential, as digital access and literacy are social determinants of health that can impact digital health use and access to care. This article discusses the potential of leveraging community Wi-Fi and spaces to improve digital access and digital health use, as well as the challenges and opportunities associated with this strategy. The existing limited evidence has shown the possibility of using community Wi-Fi and spaces, such as public libraries, to facilitate telehealth services. However, privacy and security issues from using public Wi-Fi and spaces remain a concern for librarians and healthcare professionals. To advance digital equity, efforts from multilevel stakeholders to improve users’ digital access and literacy and offer tailored technology support in the community are required. Ultimately, leveraging community Wi-Fi and spaces offers a promising avenue to expand digital health accessibility and use, highlighting the critical role of collaborative efforts in overcoming digital health disparities.

## 1 Introduction

Access to healthcare is a critical social determinant of health in the United States (US) (1, 2). Insufficient access to care can limit a person’s utilization of primary care providers, preventive care screenings, and available treatment options for chronic illnesses, leading to poorer health outcomes. Yet, access to high-quality care is constrained for many vulnerable populations in the US due to lack of health insurance, high cost of care, and proximity to healthcare facilities. In particular, access is limited among individuals residing in rural areas, individuals with lower annual income, individuals who identify as Hispanic, Black, or American Indian and Alaska Native, and immigrants with limited English proficiency (3–5). Since 2015, the proportion of US adults receiving recommended evidence-based preventive health care has decreased (6). Thus, innovative strategies are needed to overcome the barriers resulting in disparities in access to care.

Digital health, defined by the Food and Drug Administration as computing platforms, connectivity, software, and sensors for health care and related uses (7), presents a promising opportunity to improve access to care. Digital health is a “broad umbrella term encompassing eHealth (which includes mHealth), as well as emerging areas, such as the use of advanced computer sciences in ‘big data’, genomics and artificial intelligence” (8). Telemedicine is one example of digital health that has been used to improve access to care, which saw a drastic increase in use during the coronavirus disease 2019 (COVID-19) pandemic (9–11). However, disparities in digital health use exist and disproportionately burden many of the same populations experiencing barriers to accessing healthcare (10, 12). These disparities in digital health use are thought to be largely due to the digital divide, which is the gap between individuals who have access to the internet, technology, and digital literacy training and those who do not (13). According to data from the Health Information National Trends Survey (HINTS), internet access among US adults increased from 2003 (63%) to 2014 (83%) (14). However, from 2014 to 2017, internet access among US adults plateaued (14). HINTS 5 data from 2020 also demonstrated that internet access remained largely unchanged during the early stages of the COVID-19 pandemic, with 86% of US adults having internet access (15). Thus, approximately 15% of US adults are without digital access. Individuals that are older, have a lower annual household income, have lower educational attainment, and are of racial/ethnic minority groups continue to be less likely to have internet access (15, 16).

Federal programs, such as Lifeline offered by the Federal Communications Commission, provide discounts on internet service to families with incomes 135% or more below the federal poverty level or to those that participate in federal assistance programs (17). While Lifeline has the potential to help reduce national disparities in home internet access, as it is available in every state, it does not aid in paying for devices or provide strategies to improve digital literacy (17). Furthermore, a similar program - the Affordable Connectivity Program (ACP), was recently defunded by Congress which will result in approximately 1,700 internet service providers being affected and 20 million Americans struggling to afford internet access (18). The last fully funded month of ACP was April 2024 (19). Some ACP households will be eligible for the Lifeline program, but not all (19). Furthermore, not all ACP internet service providers participate in Lifeline (19). ACP participants will have the opportunity to continue their internet service from their internet company, but the cost will not be supported through ACP (19). Taken together, these federal programs, though attractive upstream solutions to address disparities in internet access, are dependent upon the political will to fund such programs. Alternative solutions, such as leveraging community Wi-Fi and spaces, are needed to promote internet and device access.

Digital access and digital literacy are increasingly being recognized as overarching social determinants of health (20–22); thus, addressing these disparities in digital equity may subsequently help to reduce disparities in digital health use and improve access to care. The purpose of this perspective piece is to discuss ways in which community Wi-Fi and spaces are used for promoting digital health access and use, as well as the challenges and opportunities associated with this strategy.

## 2 Community Wi-Fi and spaces for digital health access

The COVID-19 crisis significantly accelerated technology adoption, some say, by 7 years (23). The global count of community Wi-Fi hotspots grew from 362 million in 2019 to 549 million in 2022 (24). Places for Wi-Fi networks or hotspots have been diversified beyond school buildings, malls, hotels, or airports and can now be found in almost every public space in our neighborhoods, such as coffee shops, bakeries, hair salons, and libraries.

The existing cross-sectional and qualitative literature, from various stakeholders, provides some evidence for the potential of leveraging community Wi-Fi and spaces for digital health access, primarily public libraries for telehealth visits. For example, a survey of 39 public libraries across Virginia was conducted to determine their readiness to support telehealth visits in rural communities (25). All 39 libraries reported having internet, at least three computers, and a staff member who could answer technology questions (25). Eighty-five percent of the libraries reported having sufficient broadband speed to support audio-video telehealth visits as well as a private room from which individuals could join a telehealth visit (25). Similarly, librarians from 15 rural libraries across the US were willing to, or already did, support telehealth visits and felt it was largely aligned with their community health-focused efforts (26). Clinicians also seem supportive of telehealth visits through public library Wi-Fi. For instance, a survey of 50 nurse practitioners, physicians, and clinical nurse specialists found that the majority (82%) were supportive of patients joining telehealth visits from public libraries (27).

Despite initial evidence that public libraries may be equipped to support telehealth visits and that clinicians and librarians are largely supportive of this idea, these stakeholders have expressed concerns about the privacy and security of telehealth visits conducted from the public setting (26, 27). Additionally, librarians had concerns about individuals coming to the library when they are acutely ill instead of going to urgent care or an emergency department, potential financial burdens, and having sufficient private spaces to support telemedicine (26). We were unable to retrieve any studies evaluating patient perspectives on utilizing public libraries for telehealth visits. However, one study involving patients with type 2 diabetes revealed that the patients without digital access at home were reluctant to access the online educational portal from public spaces (e.g., public library) as they were not accustomed to visiting those venues (28).

## 3 Challenges to leveraging community Wi-Fi and spaces for digital health access

There are various challenges that may limit the use of community Wi-Fi and spaces to promote digital health access. First, some individuals may be hesitant to use Wi-Fi in community spaces to access personal information due to privacy and confidentiality concerns. In the past, using a public Wi-Fi network to get online posed certain risks to privacy because most websites did not use encryption to protect from hackers (29). However, due to the widespread adoption of encryption in recent years, connecting online through a public Wi-Fi network is now generally considered to be safe (29). Individuals that access personal health information through a public Wi-Fi network should be educated on how to tell whether their connection is encrypted (e.g., lock symbol or https next to the website address), protect their online accounts and devices, and recognize scammers to build agency around utilizing digital health (29). Providing digital literacy training and having someone available in the community space to answer questions may help to alleviate these concerns. Related, physical limitations to privacy can also be a concern when using community spaces for digital health access. For example, in the case of using a public library to join a telehealth visit, some libraries may not have the capacity or structures in place to offer a private space for individuals to connect to audio-video telehealth visits with clinicians (25, 26). Furthermore, the healthcare systems with whom patrons are connecting for telemedicine appointments may have certain privacy requirements around the transmission of patient data that may be more difficult to meet if using public Wi-Fi (30). This continues to be an evolving area of healthcare regulation.

Second, barriers to digital access can still occur even when seeking to leverage community Wi-Fi and spaces. For example, poor connectivity in some community spaces limits access to the internet making it challenging to engage with digital health platforms, including telehealth, patient portals, email, and mobile health applications (31). Public Wi-Fi tends to be slower than home broadband due to congestion from the larger number of users connecting. Additionally, rural and underserved communities may not have the same level of access to public Wi-Fi (32), meaning interventions focused on promoting the use of community Wi-Fi and spaces may not be as effective for these groups in improving healthcare access.

Third, having to travel to a community space to access Wi-Fi limits an individual’s ability to manage their health in real-time. As noted previously, patients may be reluctant to travel to public venues that they do not typically frequent or are outside their daily routine (e.g., public libraries) to utilize digital health. Thus, while leveraging community Wi-Fi and spaces may help improve digital access, digital health use, and access to care in some instances (e.g., scheduled telehealth visits) it may not be a viable option for reducing disparities in mobile health use (e.g., smartphone application for medication management). Accessing home broadband is likely a more realistic option for promoting digital health use for daily health maintenance, but public Wi-Fi can potentially be leveraged to support digital health use for scheduled healthcare appointments.

Lastly, in some populations, improving digital access may not improve uptake of digital health platforms if digital literacy is not addressed simultaneously (33). For example, older adults may need human support to navigate public Wi-Fi and utilize digital health platforms. Again, public libraries may be an opportune location for older adults to gain digital access (Wi-Fi connectivity and devices) as well as human support from library staff to successfully engage with digital health, such as scheduled telehealth visits for chronic disease management.

## 4 Discussion

The literature on leveraging community Wi-Fi and spaces for digital health is in the early stages and is very limited in size. Furthermore, available literature consists primarily of research utilizing non-experimental designs to evaluate clinician and librarian perceptions and readiness for supporting telehealth visits in public libraries, and has not investigated patient perspectives. Clinicians and librarians seem to be supportive of patients joining telehealth visits from a public library; however, there were concerns expressed mainly regarding security and privacy. Future community level program development should make sure to address these concerns. For example, small public libraries in rural settings may need additional resources to provide private spaces from which telehealth visits can be conducted.

Public libraries have undertaken other types of public health initiatives in the past to advance population health. For example, librarians have reported interacting with patrons around health and social concerns, such as housing, employment, literacy, nutrition education, and more (34, 35). Furthermore, there is interest to better enable libraries to aid in emergency and disaster preparedness and response (36). Whether it be to provide health education or improve access to care, libraries and healthcare providers should work together to meet patient needs. For example, primary care providers might provide libraries with information for patrons/patients on the benefits of physical activity and a healthy diet. Depending on the community, healthcare providers and libraries could also work together to set up private and well-equipped spaces for patients to attend telehealth visits from. Additional research in this area is warranted as it may be a promising direction for addressing disparities in digital health use. For example, patient perspectives on utilizing community spaces for digital health access should be assessed, focusing on those individuals without digital access at home or with low digital literacy. This line of research should also explore what types of digital health (e.g., telehealth, mobile health applications, electronic medical record portals) patients would be most willing to access from community spaces. Furthermore, it would be advantageous to know whether there are other community spaces, apart from public libraries, that would be considered favorable for accessing digital health platforms, especially for daily health maintenance, such as fitness and community centers, malls, senior centers, or churches. Leveraging diverse community spaces to promote digital access may be especially suitable among older adults, who generally have lower rates of digital access and literacy but are often interested in learning how to use technology if someone were to teach them (16, 37, 38).

National level survey data from US adults, in addition to the aforementioned patient perspectives, would prove useful in determining interest in leveraging community spaces for digital health access. While, to our knowledge, no national surveys currently collect this data, the Health Information National Trends Survey (HINTS) which regularly collects nationally representative data about the American public’s knowledge of, attitudes toward, and use of health-related information has the ability to assess this in the future (39). The 2022 HINTS 6 survey, is one of the first national surveys to collect data on telehealth use in the latter stages of the COVID-19 pandemic (39). Furthermore, community level experimental research is needed to evaluate whether utilizing community Wi-Fi and spaces improves digital access and digital health use among vulnerable populations.

While improving digital access is undeniably critical to reducing disparities in digital health use, it will alone not be sufficient. Improving digital literacy, often referred to as eHealth literacy (40, 41), will also be critical in some populations. Digital literacy defined as “the ability to understand and use information in multiple formats from a wide variety of sources when it is presented via computers” is crucial in a world where communication, healthcare, and access to information are increasingly reliant on technology (42). As outlined earlier, it is imperative to address the disparities in digital literacy, particularly among vulnerable populations like older adults, individuals with limited prior exposure to technology, and those residing in underserved rural areas with restricted access to digital resources (19). To promote equity in digital health use, multilevel stakeholders must prioritize the enhancement and development of digital literacy. This commitment should be shared among consumers, healthcare professionals, advocacy groups, service providers, policymakers, researchers, and industry (43).

An opportunity for improving digital equity is to offer tailored technology support services in public spaces to individuals with limited digital literacy. These services should draw upon the collective effort and engagement of the stakeholders to provide guidance, assistance, and resources necessary to build digital skills. These services should not only empower individuals to navigate digital health platforms but also instill strategies for discerning scientifically valid information from unreliable online sources. Public Wi-Fi accessibility within community spaces assumes a pivotal role in advancing this initiative, particularly for scheduled telehealth appointments addressing routine healthcare needs. Community spaces can support this by featuring interactive and simple technology such as health kiosks—publicly accessible computing devices—which have been found to be a useful tool for those with low digital literacy owing to its intuitive interface design (21, 44). Utilizing such technology may help bridge the digital divide and ensure telehealth services are accessible to a broader spectrum of the population. As the current bolster in telemedicine utilization is projected to persist, introducing digital literacy education into school curriculum is another innovative approach that can lay the groundwork for a digitally competent future generation (21). This proactive strategy not only prepares the future generation to be digitally competent but also nurtures a culture of informed healthcare consumers who can engage effectively with digital health tools.

In conclusion, digital health access in the era of post COVID-19 is at the forefront of health equity discussion. To this end, secure internet access is no longer a topic of choice but has become a fundamental necessity for many. Existing federal programs support low-cost or free internet service for low-income households, but there is an ongoing concern about sustainability as continuing such programs can be influenced by political atmosphere and/or funding availability. With adequate protection to address issues like privacy and data security, digital health access such as telehealth through community Wi-Fi and spaces, particularly public libraries, seems to present a promising and viable alternative.

## Data availability statement

The original contributions presented in the study are included in the article/supplementary material, further inquiries can be directed to the corresponding author.

## Author contributions

EMS: Conceptualization, Supervision, Writing – original draft, Writing – review & editing. HNM: Writing – original draft, Writing – review & editing. FEM: Writing – original draft, Writing – review & editing. JC: Writing – original draft, Writing – review & editing. CB: Writing – original draft, Writing – review & editing. MDH: Writing – original draft, Writing – review & editing. H-RH: Supervision, Writing – original draft, Writing – review & editing.
